# Health System Performance and Resilience in Times of Crisis: An Adapted Conceptual Framework

**DOI:** 10.3390/ijerph20176666

**Published:** 2023-08-28

**Authors:** Camille Poroes, Laurence Seematter-Bagnoud, Kaspar Wyss, Isabelle Peytremann-Bridevaux

**Affiliations:** 1 Centre for Primary Care and Public Health (Unisanté), Department of Epidemiology and Health Systems, University of Lausanne, 1010 Lausanne, Switzerland; 2 Swiss Tropical and Public Health Institute, 4123 Allschwil, Switzerland; 3 Faculty of Natural Science, University of Basel, 4001 Basel, Switzerland

**Keywords:** health systems, performance, resilience, crisis, frameworks

## Abstract

With the COVID-19 pandemic, the notion of health system (HS) performance has been discussed, and the notion of resilience has become increasingly important. Lacking a recognised framework that measures the performance of HSs throughout a crisis, i.e., one that explicitly includes time as a key aspect, we examined the literature about conceptual frameworks for measuring the performance and the resilience of HSs. This review highlighted a significant diversity among 18 distinct HS performance frameworks and 13 distinct HS resilience frameworks. On this basis, we developed a model that integrates the WHO’s widely recognised six building block framework in a novel approach derived from the European Observatory on HSs and Policies. The resulting framework adapts the building blocks to the different stages of a crisis, thereby allowing for a comprehensive assessment of an entire health system’s performance throughout the crisis’s duration, while also considering the key aspect of resilience. For a more pragmatic use of this framework in the future, indicators will be developed as a next step.

## 1. Introduction

Sanitary and environmental crises, such as those recently witnessed during the COVID-19 pandemic, may seriously impact health systems and the delivery of routine health services. The viral causing agent of SARS-Cov2 may lead to severe respiratory illnesses, the most severe cases of which can be lethal [1]. In Switzerland, as in other countries, the rapid spread and high morbidity of the virus led to overburdened emergency departments, medical wards, and intensive care services [2,3]. The usual functioning of the health system was further put under strain by the decision to prohibit elective, non-urgent examinations and surgery interventions. As a result, the medical staff in charge of COVID-19 patients faced increased activity while other services were underused [4], unable to or prohibited from providing care [5]. In this context, several publications challenged the definitions of a health system’s performance and resilience and emphasised the need for an effective public health surveillance system in order to be able to react as quickly as possible [6,7,8,9,10]. 

Globally, a health system’s performance refers to the extent to which it achieves its goals [11]. While this notion emerged several decades ago, no consensus has yet been reached on the aims of a health system. In 2000, the World Health Organization (WHO) defined the global aims of health systems as follows: to improve the health of the population, to respond to the reasonable expectations of the population, and to provide financial protection against the costs of ill health [11,12]. These global aims include more specific objectives, which may be country-specific. 

The term ‘resilience’ gained the attention of researchers on health systems during crises such as the outbreak of the Ebola virus in West Africa between 2014 and 2016 and the COVID-19 pandemic [13,14]. Until then, in Switzerland, as in other countries, it was mostly used in psychology, ecology, engineering, and materials science [15,16]. One of the multiple definitions of a health system’s resilience [17,18] was introduced by the European Observatory on Health Systems and Policies in 2020 [19]. It refers to resilience as a health system’s ability to prepare for, manage (absorb, adapt, and transform), and learn from a sudden and extreme disturbance [19]. According to Thomas et al., a crisis is a sudden and extreme change that impacts a health system. Its cycle includes four stages: stage 1: preparedness; stage 2: shock onset and alert; stage 3: shock impact and management; stage 4: recovery and learning [19]. Strengthening the capacity of health systems to be resilient is critical to effectively continue to deliver essential preventative and curative health care services to populations in times of crisis [20]. Finally, the resilience of a health system is directly linked to the surveillance system, as up to date information on the functioning of the system is a prerequisite to measure and improve resilience [21].

A resilient health system’s response to a shock requires strategies that ensure the sustained performance of a health system’s functions, thereby protecting the overall system performance [9]. Measuring the performance of a health system over time may be a relevant way of assessing how it resists a crisis, i.e., to what extent the system is resilient. Measuring the performance and resilience of health systems to understand their functioning throughout a crisis is far from easy. Indeed, since they are made of multiple domains and interactions, health systems are complex [22]. However, such measurements are crucial for preparing these systems for future shocks. Various frameworks were proposed to conceptualise and simplify the assessment of a health system’s performance or resilience [23]. However, to our knowledge, few studies combined the notions of performance and resilience, and no framework has yet conceptualised the performance of a health system in crisis situations or explicitly considered the notion of time. Lacking a recognised framework to measure the performance of health systems throughout a crisis, i.e., one that explicitly includes time as a key aspect, we studied the literature about relevant frameworks. Eventually, we adapted one of these frameworks, so it now combines both the performance and the resilience of HSs. 

## 2. Methods

### 2.1. Literature Search

A literature review was performed in May 2021, based on two distinct searches. The first one targeted health systems’ performance frameworks, while the second targeted health systems’ resilience frameworks. PubMed database was searched using the following keywords: (performan*[tiab] AND health system*[tiab] AND framework*[tiab]) for performance and (resilien*[tiab] AND health system*[tiab] AND framework*[tiab]) for resilience. In order to limit the results to performance frameworks elaborated after the well-known international frameworks issued by the WHO [24] and the Organization for Economic Cooperation and Development (OECD) [25] publication, we limited our search to the years from 2005 onwards. Regarding resilience, we used this same range of publication years because resilience is a rather new concept in the health system domain [15]. Finally, we limited our search to publications that included an abstract and were written in English or French. Given heterogeneous country-specific health systems, we focused on publications about frameworks cut out for international use and excluded papers on health system performance frameworks created specifically for a country such as Belgium, Sweden, England, Finland, Austria, and Denmark, among others [26,27,28,29,30,31,32]. We further fine-tuned our search to frameworks that encompass the overall health system. We, therefore, excluded those focusing on specific aspects of the system, such as health workforce, infectious diseases, or a pathology in particular. We then exported the result of this literature search to COVIDENCE.ORG (11 May 2021) for further consideration. In a two-step selection process, we first chose publications based on title and abstract screening, which we then fully read and selected using the eligibility criteria (Table 1). The selection was primarily completed by the first author, C.P.; L.S.B. validated it in a second step. Questionable cases were directly discussed by L.S.B. and C.P. The search was updated in April 2023.

To complement this search strategy, we screened reference lists of identified publications and grey literature (Google Scholar) and contacted several experts on the subject by e-mail, by telephone, or in face-to-face meetings. 

### 2.2. Data Abstraction

The conceptual frameworks discussed in the selected articles were analysed to identify the original model and theory on which they are based as well as their definition/objective and main dimensions/components. As suggested by Hsiao and Siadat in 2008, they were then categorised according to their descriptive, analytical, and deterministic or predictive nature [33]. While the descriptive approach informs about the components within a health system, analytical models go beyond describing what exists and further analyse some major aspects of a system and its complex operations. The deterministic or predictive models attempt to answer what factors influence the functions performed in a health system and how effective they are at doing so [34]. Finally, we evaluated these frameworks according to the following criteria: (1) international recognition: the number of articles in our literature search that were based on this model; (2) ease of use: it should allow for operationalisation (categorised as a deterministic or predictive model); (3) universality: it should apply to any health system as a whole (excluded when specific for a country or for a part of the health system, e.g., health worker specific). 

## 3. Results

### 3.1. Literature Search 

**Figure 1:**
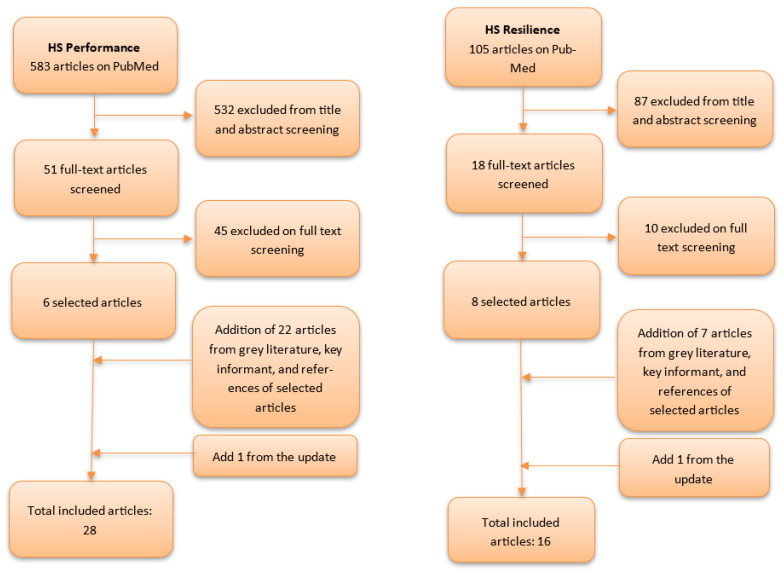
Flowchart showing the publications excluded during each step of the screening process of the literature review for publications discussing frameworks for the analysis of the performance and resilience of health systems (HS).

Figure 1 presents the flowchart of the selected literature. The search targeting performance frameworks found 583 articles. The screening of titles and abstracts selected 51 manuscripts for full-text assessment, of which 45 met one or more exclusion criteria (no framework, out of focus, specific to a national/regional health system, addressing only one part of the health system, and/or specific to a disease). This left us with six publications meeting our selection criteria (i.e., framework proposed, international scope, considers the whole health system, and covers health problems in general). We then retrieved 22 further publications found either in the grey literature, in the reference materials provided by six selected studies, following our discussions with experts, or in the update of our search. As a result, our study focused on a total of 28 publications about the performance of health systems. 

The search for publications about health system resilience frameworks retrieved 105 articles, out of which 87 were excluded based on title and abstract screening, and 10 were excluded following our full-text reading selection based on the exclusion criteria (no framework, out of focus, specific to a national/regional health system, addressing only one part of the health system, and/or specific to a disease). The remaining eight articles added to seven further articles found either in the grey literature, following discussions with experts, or in the update of our search—the latter having retrieved one publication. As a result, our study focused on a total of 16 publications about the resilience of health systems.

### 3.2. Health System Performance Frameworks

Eighteen different health system performance frameworks were found in the 28 articles of the literature review; their key features are summarised in chronological order in Table 2. These frameworks were developed between 1998 and 2022 in order to understand the functioning of health systems and have evolved thanks to the addition of specific features.

The selected frameworks are very heterogeneous. They all represent the whole or a part of a health system, either by its functions or objectives. Some of them are descriptive [35,36,37,38,39], while others have an analytical approach [12,40,41,42,43] or are more deterministic or predictive [24,44,45,46,47,48,49,50,51]. In 1998, Sicotte et al. created a framework for analysing the performance of a health system by taking into account its environment [35], which Marchal et al. fine-tuned in 2014 [49] to better assess the performance of public health organisations and to take into account the social complexity of these organisations. In 2000, the WHO first attempted to systematically measure and compare the performance of health systems [12,31] using the health system performance framework developed by Murray and Frenk (2000) [52]. The model was then adjusted in 2007 and became the six system building block framework, which breaks down the basic functions of the health system into six domains (service delivery, health workers, medical products, information, financing, and governance) [24]. Six subsequent frameworks based their own model on the WHO’s six building block model. The WHO model was augmented in multiple ways. In 2009, Don Savigny and Adam [42] added interactions. In 2017, Mfutso-Bengo et al. [38] incorporated the dimensions of leadership, ethics, governance, and systems. In 2018, Kruk et al. [43] adjusted the model for a high-quality system. In 2022, Papanicolas et al. [40] modified the WHO framework for universal health coverage, while Rohova et al. in 2017 and Fekri et al. in 2018 [31,53] also created their frameworks based on the WHO framework and on reviews. In 2006, the OECD also created a conceptual framework for the OECD’s Health Care Quality Indicator (HCQI) Project [41,54], which was used for other subsequent frameworks [31,37,40,51,55,56]. Some other authors created their own framework for a specific use, such as to reflect on health care quality (Kraft et al. in 2015 [50]), failures of a health system (the Commonwealth Fund in 2006 [44]), health sector reforms (Roberts et al. in 2008 [46]), or the context (Atun in 2010 [36]). Others built on developments in health systems’ thinking (Kruk et al. in 2008 [45]) or were primarily intended for specific settings such as the USA [47,57,58] or Canada [48]. Finally, Levesque et al. created their framework based upon a literature review in 2020, thereby seeking to bring clarity to performance assessment and avoid reductionist measures [39].

**Table 2:** Description of selected health system performance frameworks in chronological order.

Name of the Framework	Authors/Agency, Year (Ref.)	Background Theory/Original Model	Definition/Objective	Dimensions/Core Components	Categories (Descriptive, Analytical, Deterministic, or Predictive Models) *	Selection Criteria **
EGIPPS framework	Sicotte et al., 1998 [35]	Parsons’ social system action theory [59] states that every action is a product of dynamising and controlling forces, and the Competing Values Framework developed by Quinn and Rohrbaugh [60] seeks to predict whether an organisation effectively performs.	A comprehensive, theoretically grounded framework that overcomes the current fragmented approach to health care organisation performance measurement.	Organisational functions: Goal orientation (goal); Interaction with its environment to acquire resources and adapt (setting/adaptation); The integration of its internal production processes (production); The maintenance of values and norms that facilitate and constrain the previous three functions (value and culture).	Descriptive	International recognition: 1 [49] Ease of use: no Universality: no
Performance framework	WHO, 2000 [12]	Created by Murray et al. [52]	A health system includes all actors, institutions, and resources with a primary intent to improve the population’s health in ways that are responsive to the populations served and to seek to ensure a more egalitarian distribution of wealth across populations.	Four key functions of a health system determine the way inputs result in health system outcomes: resource generation, financing, service provision, and stewardship.	Analytical	International recognition: 6 [31,38,40,42,43,53] Ease of use: no Universality: yes
Six system building blocks and four outcome frameworks	WHO 2007 [24]	On the basis of the WHO 2000 [12]	Promoting a common understanding of what a health system is and what constitutes the reinforcing of health systems.	Functions: six building blocks: service delivery; workforce; information; medical products and technologies; financing; governance. Intermediate goals: access; coverage; quality; safety outcomes: improved health and health equity; responsiveness; social and financial risk protection; improved efficiency.	Deterministic and predictive	International recognition: 6 [31,38,40,42,43,53] Ease of use: yes Universality: yes
Core goals and priorities for performance improvement	Commonwealth Fund, 2006 [44]	Based on the conceptual models developed by the Institute of Medicine on quality and insurance coverage	A strategic framework for addressing the sources of system failures identified in the US health system.	A high-performance health system is designed to achieve four core goals: (1) high quality, safe care; (2) access to care for all people; (3) efficient, high-value care; (4) the system’s capacity to improve.	Deterministic and predictive	International recognition: 0 Ease of use: yes Universality: no
Conceptual framework for the OECD’s Health Care Quality Indicator (HCQI) Project	Kelley et al., 2006/Arah et al., 2006 [41,54]	Built on the dimensions of performance incorporated into a model that borrows from several previous models (US Institute of Medicine’s health care quality indicator framework; Canadian Health Indicator Framework; the WHO; the OECD)	Aims to develop a set of indicators to compare the quality of health care across OECD countries.	Focuses on objectives, particularly the quality of health care, while recognising the importance of health determinants, and health policy.	Analytical	International recognition: 5 [31,37,40,55,56] Ease of use: no Universality: yes
Systems framework	Atun et al., 2010 [36]	Built on developments in health systems thinking.	Aims to expand other HS frameworks so that they take the context into account.	System functions: the demographic, economic, political, legal and regulatory, epidemiological, socio-demographic, and technological contexts.	Descriptive	International recognition: 0 Ease of use: no Universality: yes
Framework for health systems’ performance measurement	Kruk et al., 2008 [45]	Built on developments in health systems thinking.	Aims to capture the key aspects of a health system’s functioning, to be used by policymakers and researchers.	Inputs: policies; funding; organisation. Outputs: access; quality. Outcomes: health; satisfaction; risk protection; fair financing. Dimensions of performance: effectiveness; equity; efficiency.	Deterministic and predictive	International recognition: 0 Ease of use: yes Universality: yes
Control knobs framework	Roberts et al., 2008 [46]	Developed by the World Bank Institute and the Harvard University School of Public Health.	Aims to identify areas that can be modified to strengthen health systems and improve their performance; aimed at policy makers.	Five ‘control knobs’: financing; payment; organisation; regulation; behaviour. Intermediate measures: efficiency; quality; access. Goals: health; satisfaction; risk protection.	Deterministic and predictive	International recognition: 1 [61] Ease of use: yes Universality: yes
Triple aim model (quadruple and quintuple aim)	Institute of Health Care Improvement, 2008 [47,57,58]	Developed by IHI’s innovation team.	Improving the US health care system.	The ‘Triple Aim’: improving the experience of care; improving health; reducing the cost of care for populations. They add health care workers for the quadruple aim and equity for the quintuple aim.	Deterministic and predictive	International recognition: 2 [57,58] Ease of use: yes Universality: no
Dynamic framework	Don Savigny and Adam, 2009 [42,62]	Built on the six health system; building blocks from the WHO [24].	Aims to refine the WHO framework by considering the complexity and dynamics of the health systems. Aims to allow policymakers to better design interventions and improve performance.	Adds multiple relationships and interactions across the 6 blocks, also with outcomes and goals.	Analytical	International recognition: 0 Ease of use: no Universality: yes
Health System Performance Measurement Framework	CIHI, 2013 [48]	Built on the previous CIHI—Statistics Canada Health Indicators Framework (1999).	Offers an analytical and interpretative framework that can be used to manage and improve a health system’s performance. Designed for policymakers, health system managers, and the general population.	Composed of four interrelated quadrants: social determinants of health, health system inputs and Characteristics, health system outputs, and health system outcomes.	Deterministic and predictive	International recognition: 0 Ease of use: yes Universality: no
Multipolar performance framework	Marchal et al., 2014 [49]	Modification of the EGIPPS framework [35] to better assess the performance of public health organisations and to take into account the social complexity of these organisations.	To assess the performance of a health care organisation in low- and middle-income countries by including health support organisations and infuse key elements and concepts of integrated health systems and public service to finally better deal with complexity.	The functions of the EGIPPS framework [35] are expanded to include health support organisations as well as key elements and concepts of integrated health systems and public services.	Deterministic and predictive	International recognition: 0 Ease of use: yes Universality: no
UW Health Improvement Framework	Kraft et al., 2015 [50]	Built on Donabedian’s structure-process–outcome model [63].	Aims to improve health care quality. Helpful to health system leaders.	Change domains (goals and strategies; culture; structure of learning; people, workflow, and care processes; technology) combined with levels of the health system (environment; organisation; microsystem; patients and caregivers).	Deterministic and predictive	International recognition: 0 Ease of use: yes Universality: yes
Revised OCDE framework for performance assessment	Carinci et al. OECD, 2015 [31,37,56,64]	Revised structure of the OECD framework [41,54].	Aims to build international common ground for performance measurement.	Changes to the original model: change the wording of ‘staying healthy’ to ‘primary/secondary prevention’; include the categories of ‘individual patient experiences’; ‘integrated care’ under the theme of ‘responsiveness’.	Descriptive	International recognition: 2 [51,53] Ease of use: no Universality: yes
Leadership–Ethics–Governance–System Framework (LEGS)	Mfutso-Bengo et al., 2017 [38]	Redefines the WHO’s six building blocks framework [24].	Aims to design and run a responsive and resilient health system.	The main building blocks are leadership, ethics, and governance, while the other WHO building blocks are integrated in the resilient and responsive health system element.	Descriptive	International recognition: 0 Ease of use: no Universality: no
High-quality health system framework	Kruk et al., 2018 [43]	Based on previous frameworks in the fields of health systems and quality improvement, including Donabedian [63] and the WHO [24].	Aims at high quality health systems.	Three key domains: foundations, processes of care, and quality impacts. Focuses on health system function, user experience, and how people benefit from health care.	Analytical	International recognition: 1 [61] Ease of use: no Universality: no
Integrated performance measurement framework	Levesque et al., 2020 [39]	Based on a literature review, mapping, categorisation, integration, synthesis, and validation of performance constructs.	Aims to bring clarity to performance assessment, using relevant and robust concepts and avoiding reductionist measures.	Dimensions: patients’ needs and expectations; health care resources and structures; receipt and experience of health care services; health care processes, functions, and context; outcomes. Linked to coverage, accessibility, appropriateness, effectiveness, safety, productivity, efficiency, impact, sustainability, resilience, adaptability, and equity.	Descriptive	International recognition: 0 Ease of use: no Universality: yes
Conceptual framework for health system performance assessment	Health at a Glance OCDE, 2021 [51]	Revised framework, adapted from Carinci et al. [37].	Assesses health system performance within the context of a broad view of the determinants of health.	Components of health system performance (access; quality; health system capacity; resources; subsectors, e.g., the pharmaceutical sector, ageing, and long-term care); influenced by the demographic, economic, and social context. Outcome: health status.	Deterministic and predictive	International recognition: 0 Ease of use: yes Universality: yes
HSPA Framework for UHC	Papanicolas et al., 2022 [40]	Based on the following frameworks: health systems’ performance [52]; the WHO’s building blocks [24]; control knobs [46]; the OECD’s health care quality indicators [41]; high-quality health system [43].	For universal health coverage.	Main components: functions; intermediate objectives; final goals; societal goals; while acknowledging the socioeconomic determinants of health and the political and cultural context.	Analytical	International recognition: 0 Ease of use: no Universality: yes

* As suggested by Hsiao and Siadat in 2008 [33]. ** International recognition: the number of articles in our literature search that were based on this model; ease of use: it should allow for operationalisation (categorised as a deterministic or predictive model); universality: it should apply to any health system as a whole (excluded when specific for a country or for a part of the health system, e.g., health worker specific).

### 3.3. Health System Resilience Frameworks

Based on the 16 articles identified in our literature review, we reported 13 health system resilience frameworks (Table 3). Of note, all frameworks, except the one published by Lebel et al. in 2006, were established after 2016.

Some authors gave a descriptive orientation to their framework [10,65,66,67,68,69,70,71], others used a more analytical approach (in terms of attribute or capacity) [20,72,73], while others based their framework on a deterministic and predictive approach [19,27]. Kruk et al. (2015) [74] proposed their framework in response to the demands from multilateral organisations in the aftermath of the Ebola crisis in 2014 and highlighted the five key characteristics of a resilient health system, adding a resilience index in 2017 [72]. This framework was further extended by Grimm et al. in 2021 [10], who added considerations of the strengths and weaknesses of health systems in their response to crises. Blanchet et al. (2017) [20] focused not only on the outcome of the resilience process but also on the underlying management capacities of the system and its actors to response to change. According to Fridell et al. (2019) [65], resilience reflects the ability of each of the health system’s domains to prepare, adapt, and learn from crises, be they exceptional situations or everyday challenges. Thomas et al. in 2020 [19] suggested strategies to increase the resilience of health systems. Based on the WHO framework of health systems, this model consists of four main functions of health systems, namely, governance, financing, resources, and service delivery, and considers the different phases of a crisis. In 2021, Rogers et al. [69] developed the inputs–outputs–outcomes approach, illustrating the relationships among key elements that contribute to viable and resilient health systems to support the Sustainable Development Goals. In 2022, Foroughi et al. indicated in their framework five main themes to explain and analyse the resilience of health systems, added the notion of the relationship among the phases, and changed the framework in a dynamic way [70]. Lastly, based on expert consultation, Paschoalotto et al. [71] built on previous models, in that they added important elements such as the context (including community participation) and decision making to the health system resilience framework as well as to the crisis stages model.

**Table 3:** Description of selected health system resilience frameworks in chronological order.

Name of the Framework	Authors/Agency, Year (Ref.)	Background Theory/Original Model	Definition/Objective	Dimensions/Core Components	Categories (Descriptive, Analytical, Deterministic, or Predictive Models) *	Selection Criteria **
Associations between selected attributes of governance systems and the capacity to manage resilience	Lebel et al., 2006 [73]	From social–ecological systems and ‘good governance’.	To help answer the question: how do certain attributes of governance function in society to enhance the capacity to manage resilience?	Attributes of governance: participatory; polycentric; accountable; deliberative; multilayered; fair. Capacities to manage resilience: scale; uncertainties; fit; thresholds; knowledge; diversity.	Analytical	International recognition: 1 [20] Ease of use: no Universality: no
Resilient health system framework	Kruk et al., 2017 [72,74]	Based on research and experience in health and other fields by the authors.	To measure health system resilience.	Health system resilience attributes: integrated; adaptive; self-regulating; diverse; aware.	Analytical	International recognition: 1 [10] Ease of use: no Universality: yes
Conceptual framework: the dimensions of resilience governance	Blanchet et al., 2017 [20]	Adapted from Lebel et al. 2006 [73], based on systems thinking and complexity theories.	For the analysis of health systems’ resilience.	Management capacities of the system and its actors in response to change: knowledge; uncertainties; interdependence; legitimacy. Outcomes of the resilience process: absorptive, adaptive, and transformative capacities.	Analytical	International recognition: 1 [15] Ease of use: no Universality: yes
Health system building blocks as a conceptual framework for public health disaster risk management	Olu, 2017 [27]	Based on the WHO’s six building blocks [24].	For strengthening the risk management of public health disasters.	The six building blocks are linked with other aspects: community resilience; social determinants of health; health emergency programmes; strong coordination platform; as well as with dimensions linked to health disasters (risk reduction, preparedness, emergency response, and health system recovery), to form a resilient health system.	Deterministic and predictive	International recognition: 0 Ease of use: yes Universality: no
Resilience Framework for Public Health Emergency Preparedness	Khan et al., 2018 [66]	Public health emergency preparedness theories.	To describe the essential elements of a resilient public health system and how the elements interact as a complex adaptive system.	Eleven elements including one cross-cutting element (governance and leadership) and ten distinct but interrelated elements; ethics and values at its core.	Descriptive	International recognition: 0 Ease of use: no Universality: yes
Characteristics of health system resilience within each of the WHO’s 6 building blocks	Fridell et al., 2019 [65]	Based on the WHO’s six building blocks [24].	To improve understanding of the concept of resilience.	Health system characteristics that can lead to resilience within each of the 6 building blocks.	Descriptive	International recognition: 0 Ease of use: no Universality: yes
Beyond the building blocks’ expanded framework	Sacks et al., 2019 [67]	Based on the six system building blocks from the WHO [24].	The objective of the is to expand on elements and relationships underrepresented in the dominant building block framework.	In addition to the 6 building blocks, other domains are the household production of health, social determinants of health, community organisation, and societal partnerships; outcomes are healthy people and communities.	Descriptive	International recognition: 1 [69] Ease of use: no Universality: yes
Strategies to strengthen resilience by health system function and stage in the shock cycle	Thomas et al., 2020 [19]	Based on the WHO’s building blocks [24].	To suggest strategies to strengthen resilience during the different stages of a shock cycle and for each function of a health system.	Health system function: governance, financing, resources, and service delivery; stages of a shock: preparedness; shock onset and alert; shock impact and management; recovery and learning.	Deterministic and predictive	International recognition: 0 Ease of use: yes Universality: yes
Refined Conceptual Model of Health System Resilience	Grimm et al., 2021 [10]	Based on Kruk and colleagues’ original framework [45,46].	To ascertain the relevance of health system resilience in the context of a major shock, through a better understanding of its dimensions, uses, and implications.	Five new themes were identified as foundational for achieving resilience: realigned relationships, foresight, motivation, emergency preparedness, and change management.	Descriptive	International recognition: 0 Ease of use: no Universality: yes
Determinants of health systems’ resilience framework	Haldane et al., 2021 [68]	Based on the WHO’s building blocks [24].	To review COVID-19 responses in 28 countries.	The modified building blocks are centred on community engagement as a core; they are surrounded by the notion of collaboration across sectors and health equity and outcomes.	Descriptive	International recognition: 0 Ease of use: no Universality: yes
Multidimensional Health and Social Care Systems (MHSCS) conceptual framework	Rogers et al., 2021 [69]	Builds on previous frameworks (GAVI and GFATM monitoring and evaluation framework [75], the WHO’s building blocks [24], and Sacks et al. [67]).	To illustrate the relationships among key elements that contribute to viable and resilient health systems that support the Sustainable Development Goals.	The model’s structure is based on inputs (modified building blocks), outputs (care services and intermediate goals), and outcomes (health, well-being, and financial protection), with efficiency and financing arrangement as transversal dimensions.	Descriptive	International recognition: 0 Ease of use: no Universality: yes
Health System Resiliency Analysis Framework	Foroughi et al., 2022 [70]	Based on the 6 system building blocks designed by the WHO [24], adding resilience capacities and strategies.	To create a meta-framework using the Critical Interpretive Synthesis method.	Resilience phases; intermediate objectives; goals; the WHO’s six building blocks of a health system; tools; strategies.	Descriptive	International recognition: 0 Ease of use: no Universality: yes
Health System Resilience adaptative stages and Health System Resilience framework	Paschoalotto et al., 2023 [71]	Based on the stages in Thomas et al. [19], and on the 6 system building blocks designed by the WHO [24].	Advancing towards a refinement in the health system resilience four adaptive stages model and the health system resilience framework.	The refinement includes the addition of one element considered as important by the experts, namely, the context (including community participation) as well as the importance given to decision making.	Descriptive	International recognition: 0 Ease of use: no Universality: yes

* As suggested by Hsiao and Siadat in 2008 [33]. ** International recognition: the number of articles in our literature search that were based on this model; ease of use: it should allow for operationalisation (categorised as a deterministic or predictive model); universality: it should apply to any health system as a whole (excluded when specific for a country or for a part of the health system, e.g., health worker specific).

### 3.4. Adapted Conceptual Framework Combining the Notion of Performance and Resilience

This review identified a wide range and diversity of frameworks for both performance and resilience. A few of them combined the two notions, namely, the performance and the resilience together; most used the six building blocks to feature the health system and integrated a notion of adaptation as the main feature of resilience [27,38,65,67,68,70,71]. For example, a resilient health system framework for strengthening public health disaster risk management using the six health system building blocks as basic elements was developed by Olu in 2017 [27]. In 2019, Fridell et al. [65] classified each characteristic of the resilience in the six building blocks framework developed by the WHO, and Sacks et al. [67] created the Multidimensional Health and Social Care Systems (MHSCS) conceptual framework by way of combining the elements from the six ‘building blocks’ framework (WHO). Haldane et al. in 2021 [68] developed a resilient health systems framework based on the WHO’s health systems building blocks framework, with community engagement as core to all elements of health systems’ resilience. Similarly, Paschoalotto et al. [71] also proposed a health system resilience framework based on the WHO’s health systems building blocks and added several elements: the context, communication, and social participation, as well as decision makers as a core component. Finally, Foroughi et al. [70] added the phases of resilience, resilience strategies, and resilience tools to the health system building blocks model. However, none of them introduced the notion of resilience in a health system performance framework as its changes over time, which would allow for assessment in times of crisis.

**Figure 2:**
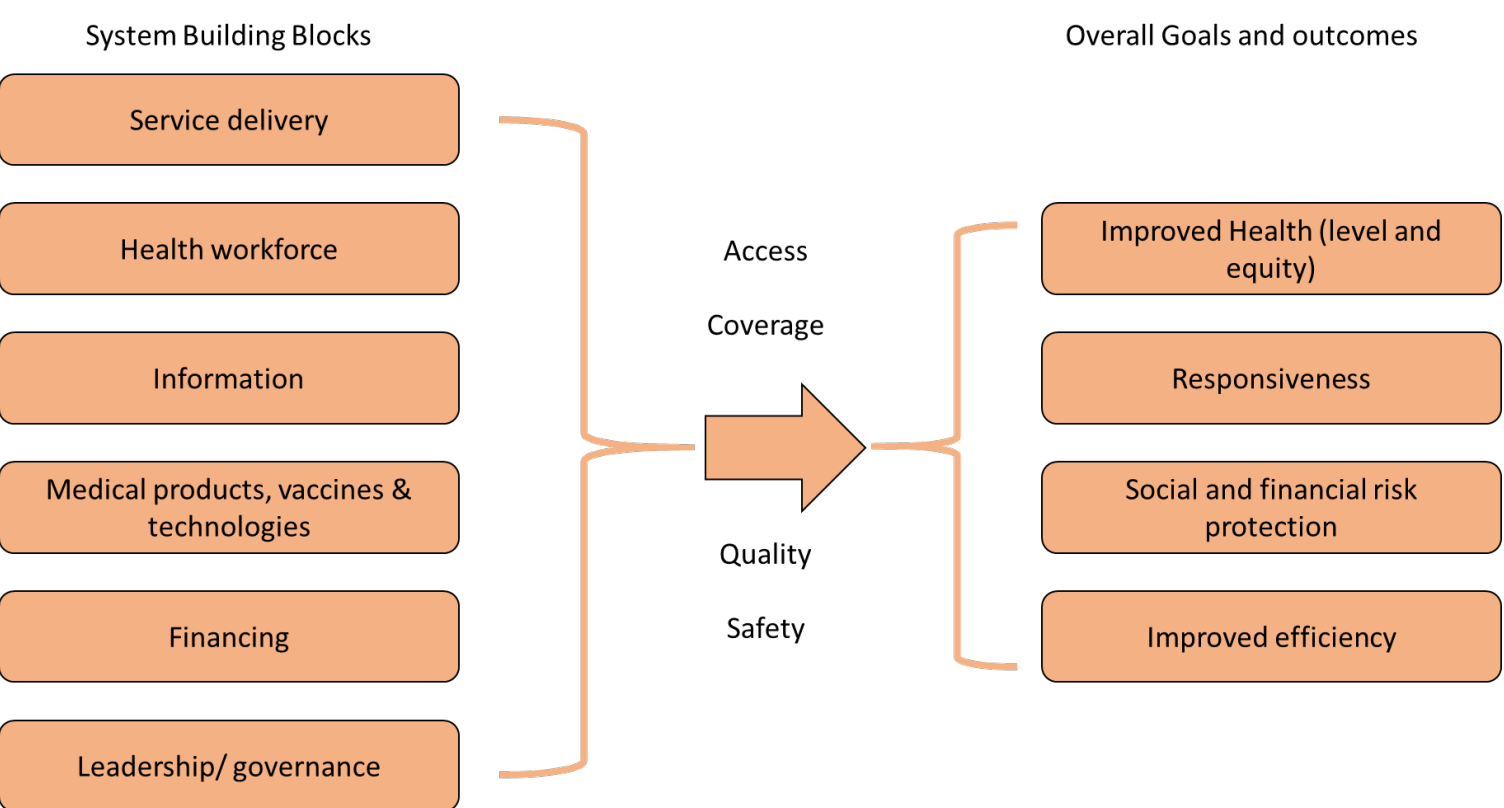
The six system building block and four outcome framework (an adapted version of the WHO’s health system framework, 2007 [24]).

Thus, following the selection criteria—i.e., international recognition, ease of use, and universality—and out of the selected existing health system performance frameworks, the WHO’s six building block model (Figure 2) appeared as the best-suited framework for combining the performance and resilience of health systems. Indeed, it is the most internationally recognised framework and has undergone several iterative versions, up to the last model published in 2007 [24]. Our literature review showed that this framework was often used as a foundation for subsequent frameworks and that some authors adjusted it to meet the needs of their particular research, even in recent articles on resilience [62,70]. The WHO framework is the most frequently cited model just ahead of the OECD’s HCQI framework [41,54]. This model has been criticised, as it assumes that each block is of equal importance and does not explicitly include the interactions between the blocks as well as the interactions among the input, output, and outcome. However, according to the WHO, to achieve its goals, a health system must first build on these six ‘basic’ functions, all of which are necessary to improve performance [75] and sustain improvements in health [76]. Furthermore, this model has some incontestable advantages that meet our selection criteria, as it is internationally recognised, is easy to apply in the sense that it allows for operationalisation, is universal, is suited to every health systems, and creates a common language and shared understanding [77]. The building blocks approach is a useful tool for locating, describing, and classifying health system constraints and for identifying where and why investments are needed, what will happen as a result, and by what means the change can be monitored [34].

**Figure 3:**
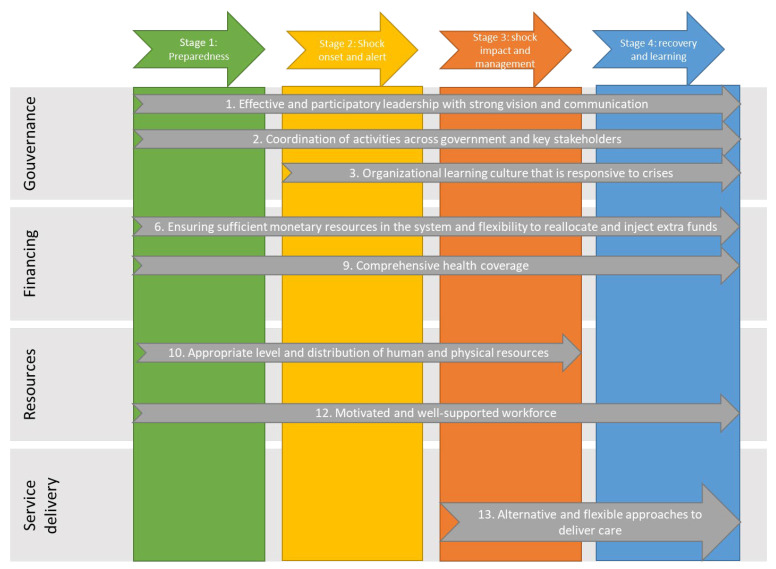
Selected strategies to strengthen resilience by health system function and stage in the shock cycle (adapted from Thomas et al. (2020) [19]).

Although the WHO recognised that the resilience of health systems plays a critical role in global health and sustainable development [78], it did not explicitly integrate this notion into its six building block framework. In order to do so, we chose to use the approach developed by the European Observatory on Health Systems and Policies by Thomas et al. [19] (Figure 3), which was selected in the literature review about resilience frameworks according to our selection criteria (international recognition, ease of use, and universality). Recurringly quoted in recent articles on the subject [69,70,79], this model is based on the WHO’s previous framework but additionally considers that resilience may vary during the different stages of a crisis, making it possible to conceptualise change over time.

**Figure 4:**
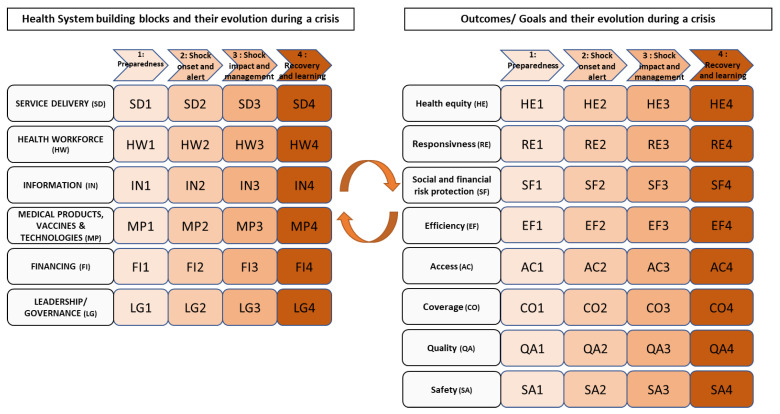
Health system performance and resilience framework, by the authors.

Consequently, and so reflecting both performance and resilience considerations, our model combined these two models (Figure 4). Each of the six building blocks identified by the WHO and the intermediate and final outcomes and goals that were merged at the same level are developed according to the different stages of a crisis. Each specific block (e.g., SD1, SD2, and HW1) represents the state of a building block or an outcome/goal for a specific phase of the crisis, knowing that stage 1 represents the ‘preparedness’, stage 2 the ‘shock onset and alert’, stage 3 the ‘shock impact and management’, and stage 4 the ‘recovery and learning’. We added arrows to note that the health system is dynamic and teems with interactions among and across all building blocks, as Don de Savigny and Adam noticed in their refined framework [42,62]. These bidirectional arrows are in the centre of the framework, between the building blocks and the goals and outcomes, because of a continuous adjustment between the input and output. Thus, our framework conceptualises the performance of the whole health system over time during a crisis by combining the WHO’s six building block performance framework [24] with the resilience approach developed by Thomas et al. [19].

## 4. Discussion

This literature review allowed for the identification of 18 HS performance and 13 HS resilience frameworks. While showing the latter’s wide range and diversity, an important finding of this review is that the two distinct literature searches did not retrieve the same frameworks. This means that the notion of performance and resilience are two different concepts with their own specific definition, their own representation, and their own body of literature. Also, while a few frameworks linked the two notions in some ways, and a few used the six building blocks as a foundation [27,38,65,67,68,70], none of them declined each building block into the various phases of a crisis.

Health system performance frameworks tend to be static and do not reflect actions, decision points, or change over time, for example, in times of crisis. This static nature may explain why the resilience frameworks, which are expected to reflect change over time and the dynamic nature of a crisis, do not refer to the health system performance frameworks. In that sense, our model can serve as a basis for measuring the performance of health systems in a more crisis-sensitive manner, with goals that are suited to the situation in which the health system finds itself. Our model follows the structure of the WHO’s six building blocks. Its application may be criticised on the grounds that the building blocks appear to be equally important from a visual point of view. However, declining each building block into crisis phases could be a first step towards improving the model.

In addition, the literature review indicates that research on how to quantitatively measure resilience and how it is related to the performance of a health system is limited. This may be related to the complexity of the measurements of health systems globally and time trends [80]. The quantitative metrics that exist do not refer to resilience, so shocks and their effects can hardly be assessed along the phases of a crisis. Therefore, our model is a first step towards considering resilience as an important dimension of performance. Indeed, since our model combines rather quantitative aspects of performance with rather qualitative aspects of resilience, it offers a way of understanding the resilience of a health system from both a qualitative and a quantitative point of view. Quantitative data are needed throughout crises to monitor the variation in performance over time, an important element to assessing the resilience of a health system.

Almost all the health system resilience frameworks reported in this review were established after 2016, a sign of growing interest in this topic since that year. Moreover, most of them are merely descriptive. This result reveals a lack of knowledge about how these frameworks can be applied in practice (as opposed to merely used for description), in view of increasing the resilience of health systems. According to the work of Forsgren et al. in 2022, the recovery and learning aspects mostly lack future resilience [21]. This highlights the need for an adapted framework that has the potential for operationalisation and goal setting. In that sense, this adapted framework is a tool that could be used by health information systems in public health surveillance. A further step towards using this framework on an operational basis would require complementing this framework with performance and resilience indicators specific to each block. These would make it possible to assess the performance of a health system not only during a crisis but also before and after a crisis and, thus, evaluate its resilience. It would also allow for comparing results between health systems. There is currently room for improvement in the monitoring of current health systems’ performance, particularly as far as times of crisis are concerned. Its improvement is crucial when it comes to building resilient health systems and providing adequate health services in times of crisis [81]. A set of performance and resilience indicators covering all the domains of a health system, with specific targets according to time phases, is still needed to assess performance over time and eventually create resilient systems. A next step of our work will be to identify and select such indicators.

## 5. Conclusions

Based on a contemporary literature review, we propose an adjusted framework that serves as a practical tool to facilitate the assessment of the performance and resilience of a health system in times of crisis. Heavily influenced by the COVID-19 pandemic and the emergence of health system resilience frameworks, it is also more relevant globally, in times of crises; also, it is practical for identifying key issues that the health system may face in a crisis. Finally, by identifying areas of improvement, it can be useful for health system stakeholders to inform their decision makers and develop strategies.

## Figures and Tables

**Table 1:** Eligibility criteria for publications.

**Inclusion Criteria**
Published since 2005
Full text in English or French
With an abstract
**Exclusion Criteria**
Presenting no framework
Out of focus
Specific to a national/regional health system
Addressing only one part of the health system
Specific to a disease

## Data Availability

Not applicable.
